# Overcoming Hypoxia-Mediated Tumor Progression: Combinatorial Approaches Targeting pH Regulation, Angiogenesis and Immune Dysfunction

**DOI:** 10.3389/fcell.2016.00027

**Published:** 2016-03-31

**Authors:** Paul C. McDonald, Shawn C. Chafe, Shoukat Dedhar

**Affiliations:** ^1^Department of Integrative Oncology, British Columbia Cancer Research CentreVancouver, BC, Canada; ^2^Department of Biochemistry and Molecular Biology, University of British ColumbiaVancouver, BC, Canada

**Keywords:** hypoxia, tumor microenvironment, carbonic anhydrase IX, monocarboxylate transporter, angiogenesis, immune checkpoint inhibitors

## Abstract

Hypoxia is an important contributor to the heterogeneity of the microenvironment of solid tumors and is a significant environmental stressor that drives adaptations which are essential for the survival and metastatic capabilities of tumor cells. Critical adaptive mechanisms include altered metabolism, pH regulation, epithelial-mesenchymal transition, angiogenesis, migration/invasion, diminished response to immune cells and resistance to chemotherapy and radiation therapy. In particular, pH regulation by hypoxic tumor cells, through the modulation of cell surface molecules such as extracellular carbonic anhydrases (CAIX and CAXII) and monocarboxylate transporters (MCT-1 and MCT-4) functions to increase cancer cell survival and enhance cell invasion while also contributing to immune evasion. Indeed, CAIX is a vital regulator of hypoxia mediated tumor progression, and targeted inhibition of its function results in reduced tumor growth, metastasis, and cancer stem cell function. However, the integrated contributions of the repertoire of hypoxia-induced effectors of pH regulation for tumor survival and invasion remain to be fully explored and exploited as therapeutic avenues. For example, the clinical use of anti-angiogenic agents has identified a conundrum whereby this treatment increases hypoxia and cancer stem cell components of tumors, and accelerates metastasis. Furthermore, hypoxia results in the infiltration of myeloid-derived suppressor cells (MDSCs), regulatory T cells (Treg) and Tumor Associated Macrophages (TAMs), and also stimulates the expression of PD-L1 on tumor cells, which collectively suppress T-cell mediated tumor cell killing. Therefore, combinatorial targeting of angiogenesis, the immune system and pH regulation in the context of hypoxia may lead to more effective strategies for curbing tumor progression and therapeutic resistance, thereby increasing therapeutic efficacy and leading to more effective strategies for the treatment of patients with aggressive cancer.

## Introduction

The tumor microenvironment (TME) is heterogeneous in its composition and dynamic in its evolution. Hypoxia is a prominent component of the TME of solid cancers and is a result of an imbalance between the increasing demand for oxygen and nutrients by rapidly proliferating tumor cells and an inadequate, dysfunctional blood supply resulting from tumor angiogenesis (Lendahl et al., 2009; Bailey et al., 2012). The presence of hypoxia is an independent marker of poor prognosis in many types of human cancer, including breast, non-small cell lung, head and neck, ovarian and cervical cancer (Brahimi-Horn et al., 2007; Semenza, 2014). Furthermore, hypoxia is a functional contributor to several biological processes critical for cancer progression, including angiogenesis, epithelial-mesenchymal transition (EMT), migration/invasion, maintenance of cancer stem cells (CSCs) and the associated CSC niche, metastasis, immune surveillance and resistance to chemotherapy and radiotherapy (Gillies et al., 2012; Parks et al., 2013). Thus, it is not surprising that hypoxia is associated with reduced patient survival in many cancers (Semenza, 2014). Importantly, the development of hypoxia in the TME produces substantial cellular stress that drives adaptive responses by cancer cells aimed at providing advantages for survival, growth and metastasis (Gatenby and Gillies, 2008; Kroemer and Pouyssegur, 2008; Damaghi et al., 2013; Marchiq and Pouysségur, 2015). Targeting the molecular machinery necessary for driving these adaptations is a critical strategy required for the development of effective cancer therapy.

### Role of metabolic reprogramming and angiogenesis in pH dysregulation

Early in tumor development, cancer cells proliferate beyond the limit of diffusion and confront hypoxia, resulting in the inhibition of energy production by oxidative phosphorylation. The increasing demand for energy in the form of ATP and for anabolic precursors by rapidly proliferating cancer cells in this low oxygen environment triggers rapid metabolic reprogramming (Marchiq and Pouysségur, 2015). Tumor cells undergo a “metabolic switch” and, through activation of HIF-1α, shift toward increased use of glycolysis to efficiently maintain cellular bioenergetics and macromolecular biosynthesis in increasingly restrictive growth conditions (Pouyssegur et al., 2006; Marchiq and Pouysségur, 2015). As a result of metabolic reprogramming, cancer cells display increased heterogeneity in glucose metabolism, investing most heavily, but not exclusively, in glycolysis, while continuing to utilize glucose oxidation, albeit at a reduced level in hypoxic regions (Sonveaux et al., 2008; Hensley et al., 2016; Pavlova and Thompson, 2016). Furthermore, it is now recognized that cancer cells use alternative carbon sources, especially glutamine, to promote the tricarboxylic acid (TCA) cycle (Sonveaux et al., 2008; Hensley et al., 2016; Pavlova and Thompson, 2016). The perpetuation of a level of oxidative phosphorylation in the hypoxic microenvironment further reduces the amount of oxygen available, effectively exacerbating hypoxia (Sonveaux et al., 2008; Hensley et al., 2016). Importantly, glycolytic metabolism is associated with increased metabolic plasticity, allows cancer cells to utilize glucose for both energy and biosynthesis (Payen et al., 2015a,b), and fuels the “Warburg effect” in which cancer cells maintain highly glycolytic metabolism even in the presence of oxygen (Marchiq and Pouysségur, 2015). However, a major consequence of the switch to glycolytic metabolism by hypoxic cancer cells is the production of acidic metabolites, including lactate and protons (H^+^), which result in increased tumor acidosis and further challenge cell survival (Brahimi-Horn et al., 2011; Webb et al., 2011; Damaghi et al., 2013; Gillies and Gatenby, 2015).

In addition to regulating metabolic reprogramming, the HIF-1-mediated transcriptional program induced by cancer cells in hypoxia drives the process of tumor angiogenesis, a hallmark of cancer progression (Hanahan and Weinberg, 2011). Angiogenesis may be viewed as an initial attempt by the growing tumor to alleviate hypoxia and provide vital nutrients and oxygen to cancer cells (Kerbel, 2008; Rapisarda and Melillo, 2012; Welti et al., 2013; McIntyre and Harris, 2015). However, in contrast to the balanced, tightly regulated multistage process of angiogenesis in normal tissues, tumor angiogenesis is aberrantly regulated and leads to vessels that are tortuous, leaky and dysfunctional (Kerbel, 2008; De Bock et al., 2011; Rapisarda and Melillo, 2012; Welti et al., 2013). As a result, tumor tissues generally exhibit poor perfusion and increased interstitial pressure, a situation that leads to significant biological consequences, including the further development of hypoxic regions, a reduction in nutrient delivery and a reduced ability to remove rapidly accumulating acidic metabolites from the TME (Gillies et al., 2012; Rapisarda and Melillo, 2012). Therefore, the switch to glycolytic metabolism by cancer cells and the angiogenic response to tumor hypoxia collude to create an increasingly acidic, hypoxic TME that fuels adaptations by tumor cells that are geared toward enhanced survival and growth in an otherwise hostile environment.

## Targeting pH regulation and hypoxia-driven acidosis

A major ramification of the generation of large amounts of acidic metabolites by glycolytic cancer cells, coupled with impaired perfusion and diffusion capacity in hypoxia, is increasing intracellular and extracellular acidosis (Webb et al., 2011; Damaghi et al., 2013; McIntyre and Harris, 2015). The accumulation of lactate and H^+^ in glycolytic cells, if left unbuffered, leads to intracellular acidification and apoptosis (Webb et al., 2011; Damaghi et al., 2013), while a slightly alkaline intracellular pH (pHi) is permissive for proliferation (Webb et al., 2011). In contrast, acidification of the TME stimulates breakdown of the extracellular matrix, and promotes migration, invasion and metastasis (Webb et al., 2011; Damaghi et al., 2013). Therefore, to both mitigate the potentially lethal consequences of increasing intracellular acidosis and exploit the advantages of a hypoxic, acidic TME, cancer cells upregulate the molecular machinery necessary to maintain a reverse pH gradient (i.e., alkaline pHi and acidic pHe) that acts to promote survival, proliferation, invasion and metastasis (Webb et al., 2011; Damaghi et al., 2013). Key pH regulatory components that cancer cells upregulate in hypoxia include membrane-bound, extracellular carbonic anhydrases (CAs), particularly CAIX and CAXII, which maintain an intracellular and extracellular acid-base balance (McDonald et al., 2012; Damaghi et al., 2013; McDonald and Dedhar, 2014), and monocarboxylate transporters (MCTs), especially MCT4, which facilitate lactate extrusion (Marchiq and Pouysségur, 2015). The importance of these factors in regulating pH in hypoxia has resulted in their exploitation as therapeutic targets across a broad spectrum of solid tumors, as discussed below.

### Carbonic Anhydrase IX

**Figure 1:**
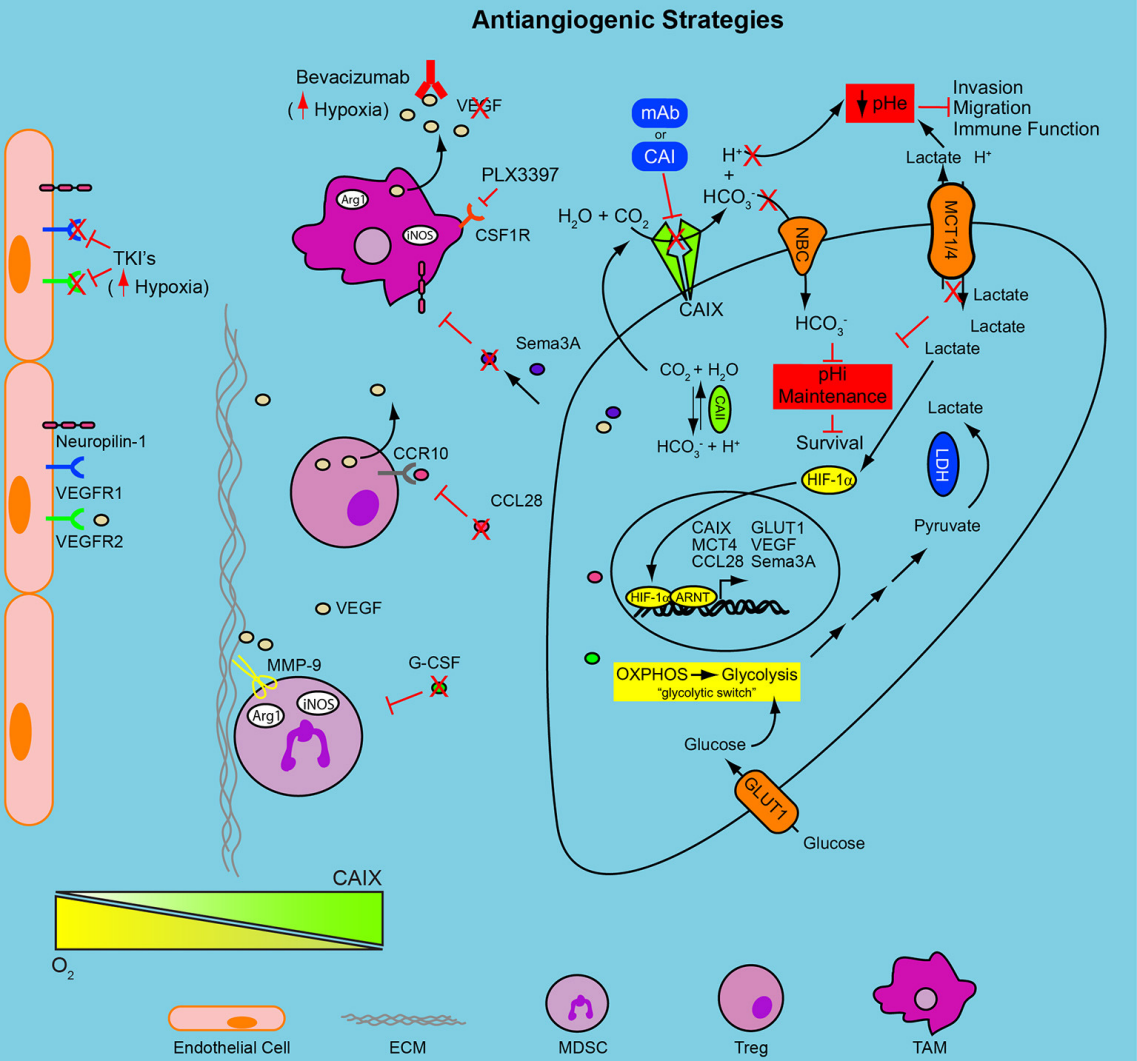
**Combinatorial approaches to target the hypoxic TME and anti-angiogenic resistance**. Hypoxia induces a HIF-1-mediated signaling cascade that results in nuclear translocation of HIF-1α and activation of hypoxia-regulated genes, including GLUT1, MCT4, and CAIX. The decreased oxygen availability forces tumor cells to become reliant upon glycolysis for energy production, the “glycolytic switch”; the concomitant accumulation of intracellular glycolytic byproducts forces the upregulation of transporters, MCT1/4, to cope with the declining pHi, thus acidifying the extracellular environment. The upregulation of CAIX contributes to the decreasing pHe, through the production of H^+^, and to the regulation of pHi through the production of ${\text{HCO}}_{3}^{-}$, which re-enters the cell and buffers intracellular acidosis. The declining pHe activates proteases, increasing migration and invasion and reduces immune function. The use of anti-angiogenic drugs, tyrosine kinase inhibitors or VEGF antibodies (Bevacizumab and TKI's), increases the hypoxic fraction of the tumor and engages the HIF program. This adaptation may then render those cancer cells vulnerable to the metabolic adaptations initiated above. Blocking the pH regulatory machinery, CAIX and MCTs, may be an effective combinatorial strategy for reducing tumor cell survival and overcoming resistance to anti-angiogenic therapy. Stromal cell populations (MDSC, TAM, and Treg) recruited to the hypoxic niches of solid tumors contribute to resistance to anti-angiogenic therapy. Limiting their recruitment to the TME by reducing circulating levels of soluble factors (e.g., G-CSF, CCL28) or antagonizing cellular receptors (PLX3397) needed for their chemotaxis in combination with the modalities mentioned above may reduce resistance to anti-angiogenic therapy and improve therapeutic outcome. pHe, extracellular pH; pHi, intracellular pH; mAb, monoclonal antibody; CAI, carbonic anhydrase inhibitor; ${\text{HCO}}_{3}^{-}$, bicarbonate; H^+^, proton; CO_2_, carbon dioxide; H_2_O, water; NBC, sodium/bicarbonate cotransporter; MCT1/4, monocarboxylate transporter 1 and 4; HIF-1α, hypoxia-inducible factor 1 alpha; ARNT, aryl hydrocarbon receptor nuclear translocator; LDH, lactate dehydrogenase; OXPHOS, oxidative phosphorylation; CAII, carbonic anhydrase II; CAIX, carbonic anhydrase IX; GLUT-1, glucose transporter 1; Sema3A, semaphorin 3A; VEGF, vascular endothelial growth factor; CCL28, chemokine (C-C motif) ligand 28; CCR10, chemokine (C-C motif) receptor 10; Arg1, arginase 1; iNOS, inducible nitric oxide synthase; CSF1R, colony stimulating factor 1 receptor; TCR, T cell receptor; G-CSF, granulocyte colony stimulating factor; TAM, tumor associated macrophage; MDSC, myeloid-derived suppressor cell; Treg, regulatory T cell; ECM, extracellular matrix; MMP9, matrix metalloprotease 9; VEGFR1, vascular endothelial growth factor receptor 1; VEGFR2, vascular endothelial growth factor receptor 2; TKI, tyrosine kinase inhibitor.

Carbonic Anhydrase IX (CAIX) is a major effector of the HIF-1-mediated transcriptional response to tumor hypoxia and its critical role in tumor progression is well-recognized (McDonald et al., 2012; McDonald and Dedhar, 2014; Pastorek and Pastorekova, 2015). It is highly expressed in the hypoxic regions of many types of solid tumors, has a very restricted expression profile in normal tissues and is a well-established marker of poor prognosis across a wide spectrum of solid cancers (McDonald et al., 2012; Pastorek and Pastorekova, 2015). Of importance for its utility as a cancer therapeutic target, CAIX is a critical, hypoxia-induced functional effector of several biological processes necessary for cancer growth and metastasis, including pH regulation and cell survival, migration and invasion, maintenance of cancer stem cell (CSC) function, development of the pre-metastatic niche and acquisition of chemo and radioresistant properties (McDonald et al., 2012; McDonald and Dedhar, 2014; Chafe and Dedhar, 2015; Pastorek and Pastorekova, 2015). By catalyzing the reversible hydration of CO_2_ to bicarbonate (${\text{HCO}}_{3}^{-}$) and protons (H^+^) at the extracellular surface (Gillies et al., 2008; McDonald et al., 2012; Parks et al., 2013; Sedlakova et al., 2014), CAIX controls an intracellular and extracellular acid-base balance that regulates both survival and invasive properties (Figure 1). The ${\text{HCO}}_{3}^{-}$ produced by CAIX re-enters the cell through bicarbonate transporters and anion exchangers, thereby buffering intracellular acidosis and facilitating tumor cell survival and growth. The H^+^ participate in the generation of an increasingly acidic extracellular environment, a phenomenon recently demonstrated in models of colorectal cancer *in vivo* using hyperpolarized ^13^C-magnetic resonance spectroscopy (Gallagher et al., 2015), fueling the breakdown of the extracellular matrix and facilitating tumor cell invasion and metastasis (Swietach et al., 2010; McDonald et al., 2012; Parks et al., 2013; Sedlakova et al., 2014). Congruent with its role in regulating pH, several studies have demonstrated that perturbing CAIX function in hypoxia elicits biological consequences that impede cancer progression and demonstrate its utility as a therapeutic target.

Stable depletion of CAIX expression or inhibition of its activity with small molecule inhibitors (discussed in detail below) in the context of hypoxia results in the inhibition of tumor growth across multiple models, including breast cancer (Lou et al., 2011), colorectal cancer (Chiche et al., 2009; McIntyre et al., 2012) and glioblastoma (McIntyre et al., 2012), and demonstrates a critical role for CAIX in cancer cell survival *in vivo*. Perturbation of CAIX function in hypoxia also blunts migration and invasion of cancer cells *in vitro* (Proescholdt et al., 2012; Lock et al., 2013), and inhibits the formation of metastases *in vivo* (Lou et al., 2011; Gieling et al., 2012). Importantly, the role of CAIX in migration, invasion and metastasis is linked to its catalytic activity and the production of H^+^, which helps to drive development of acidosis within the extracellular environment and facilitates local invasion through disruption of the extracellular matrix, activation of metalloproteases and increased cell invasiveness (Estrella et al., 2013; Svastova and Pastorekova, 2013; Sedlakova et al., 2014; Pastorek and Pastorekova, 2015). Furthermore, evidence now strongly suggests that CAIX is an integral functional component of CSCs. Several studies have shown that CAIX is required for stemness properties of CSCs in hypoxia (Lock et al., 2013; Papi et al., 2013; Ledaki et al., 2015; Pore et al., 2015), and treatment of orthotopic human breast cancer xenografts with specific small molecule inhibitors of CAIX significantly reduced the CSC population. Increased CAIX expression was also observed in the tumor initiating cell fraction of pancreatic ductal adenocarcinoma in a patient-derived xenograft cell line and targeting CAIX expression in this population of cells with shRNA greatly reduced their tumor initiating capacity (Pore et al., 2015). Together, these studies demonstrate a functional role of CAIX in maintenance of the CSC population *in vivo* and suggest that pharmacologic targeting of CAIX may be effective at reducing or eliminating CSCs in hypoxia, a cell population that is resistant to conventional chemotherapy and radiotherapy. These attributes, together with its ease of accessibility to pharmacologic agents due to its membrane-bound, extracellular catalytic domain, have made CAIX a very attractive target for cancer therapy (Neri and Supuran, 2011; Wilson and Hay, 2011; McDonald et al., 2012; Supuran, 2012; Pastorek and Pastorekova, 2015).

It is clear from the discussion above that therapeutic targeting of CAIX holds potential for enhanced treatment efficacy through the elimination of aggressive cancer cells that have adapted to hypoxia, a realization that has spawned extensive efforts to develop therapeutics targeting CAIX. Two overarching and complementary approaches have been utilized to target CAIX for cancer treatment. One approach has involved the development of therapeutic modalities, especially CAIX-specific small molecule inhibitors that directly target the catalytic activity of CAIX, thereby exploiting the biological roles of CAIX, including pH regulation, migration and invasion, and CSC maintenance (Figure 1). A second, complementary approach exploits the tumor-specific upregulation of CAIX as a highly selective “address” to which to deliver CAIX-specific monoclonal antibodies, either alone or in combination with cytotoxic or radioactive warheads to elicit killing of hypoxic tumor cells. Both of these approaches are discussed below.

#### Small molecule inhibitors

The development of small molecule inhibitors of CA activity that are selective for cancer associated extracellular CAs such as CAIX (and CAXII) over other, closely related “off-target” CA isoforms has been an area of intense investigation during the past few years (Neri and Supuran, 2011; Supuran, 2012). These studies have resulted in a large number of novel, potent CAIX-selective inhibitory compounds, some of which are now being evaluated *in vivo* and in the clinic (McDonald et al., 2012; McDonald and Dedhar, 2014). While initial strategies to enhance CAIX selectivity of the pan CA inhibitor, acetazolamide, were met with limited success (Ahlskog et al., 2009; Chiche et al., 2009), a series of novel, potent, CAIX-selective “next generation” small molecule inhibitors are showing great promise. Several classes of novel CAIX inhibitors, including ureidosulfonamides (Pacchiano et al., 2011), glycosyl coumarins (Touisni et al., 2011) and indanesulfonamides (Dubois et al., 2011) have been used successfully to inhibit tumor growth in preclinical models of hypoxic, CAIX-positive breast cancer (Lou et al., 2011; Pacchiano et al., 2011; Touisni et al., 2011) and colorectal cancer (Dubois et al., 2011), demonstrating that the selective pharmacologic inhibition of CAIX activity elicits an anti-tumor effect *in vivo*. Furthermore, the ureidosulfonamide and glycosyl coumarin inhibitors were effective in reducing lung metastases (Lou et al., 2011; Pacchiano et al., 2011), as was a sulfamate inhibitor of CAIX (Gieling et al., 2012), and in depleting CSCs in models of breast cancer metastasis (Lock et al., 2013), showing the value of CAIX inhibitors as therapeutic agents for use in targeting metastasis and chemoresistant tumor initiating cells.

These exciting data and further assessment of lead compounds resulted in the selection of a novel, first in class, highly selective ureidosulfonamide inhibitor of CAIX and CAXII, SLC-0111 (aka U-104; Lou et al., 2011; Pacchiano et al., 2011), for clinical development. Pharmacokinetic and toxicology studies demonstrated negligible toxicity and >10-fold therapeutic index, and SLC-0111 is orally bioavailable. SLC-0111 is now the subject of a first in man, multi-center phase 1 clinical trial (NCT02215850) in patients with solid tumors and is nearing completion of recruitment (Table 1). In addition to this pivotal clinical trial, data demonstrating preclinical efficacy of another CAIX inhibitor/radiosensitizer, DTP-348, in breast and colorectal models alone and in combination with either ionizing radiation (Dubois et al., 2013; Ward et al., 2015) or doxorubicin (Rami et al., 2013) has resulted in reports of a phase 1 clinical trial (NCT02216669) in patients with solid tumors (McIntyre and Harris, 2015; Table 1). However, this study is not currently open for recruitment of patients. Importantly, if clinical trials utilizing inhibitors of CAIX activity are to be successful, selection of those patients likely to respond to therapy is critical. Only patients whose tumors require CAIX function for survival, growth and metastasis are likely to benefit. Thus, predictive biomarkers, including CAIX expression and additional measures of hypoxia response, will be necessary to guide patient selection for these therapies.

**Table 1:** **Clinical trials targeting pH regulators in cancer**.

**Target**	**Drug**	**Intervention**	**Phase**	**Identifier**
CAIX	SLC-0111	Advanced solid tumors	1	NCT02215850
CAIX	Girentuximab (cG250)	Kidney Cancer	3	NCT00087022
CAIX	DTP-348	Solid tumors	1	NCT02216669
CAIX	^177^Lu-cG250	Metastatic RCC	2	NCT00142415
CAIX	AdGMCAIX transduced DC	Metastatic RCC	1	NCT01826877
MCT1	AZD3965	Solid tumors, Gastric and prostate cancer, DLBCL	1	NCT01791595

*DC, dendritic cell; RCC, renal cell carcinoma; DLBCL, diffuse large B cell lymphoma*.

#### Therapeutic antibodies against CAIX

Immunotherapy using CAIX-specific monoclonal antibodies (mAbs) may derive its therapeutic efficacy through several mechanisms (McDonald et al., 2012; Pastorek and Pastorekova, 2015) (Figure 1). For example, direct binding of the mAb to CAIX can elicit an anti-tumor response due to antibody-mediated cell cytotoxicity (ADCC). Alternatively, high affinity CAIX mAbs capable of receptor-mediated internalization have the potential to provide effective vehicles for targeted delivery of various therapeutic compounds, including cytotoxins and radionuclides, as has been demonstrated clinically for antibody-drug conjugates such as ado-trastuzumab emtansine (T-DM1) in breast cancer (Lambert and Chari, 2014). Thus, the development of CAIX-specific therapeutic mAbs is an active area of research and one with potential to yield important advances in cancer therapy.

Girentuximab (cG250) is arguably the most clinically advanced mAb against CAIX, particularly for renal cell carcinoma (Oosterwijk, 2008; Oosterwijk-Wakka et al., 2013). Initial studies demonstrated that cG250 could elicit antibody-dependent cellular cytotoxicity (ADCC) (Surfus et al., 1996), an established mechanism by which therapeutic mAbs function to destroy tumor cells. This property of therapeutic mAbs is well-established clinically, and a recent study has demonstrated that modification of the Fc region of a CAIX mAb originally identified in a high throughput screen (Xu et al., 2010) increased ADCC *in vitro* and was effective at targeting orthotopic RCC tumors in an immunocompromised mouse model following allogeneic transplantation of human peripheral blood mononuclear cells (Chang et al., 2015). Girentuximab is marketed by WILEX AG under the trade name RENCAREX® and Phase I and II trials demonstrated that this Ab was safe, well-tolerated and able to positively impact disease burden, alone and together with IL-2 treatment (Davis et al., 2007; Zatovicova et al., 2010; Neri and Supuran, 2011; Siebels et al., 2011). However, a phase 3 trial (ARISER; NCT00087022) targeting patients with non-metastatic renal cell carcinoma failed to show an improvement in disease-free survival with treatment (Pastorek and Pastorekova, 2015). It should be noted that this study lacked stratification of patients based on CAIX expression which, if accounted for, showed significant improvement in the subset of patients with high CAIX expression, demonstrating the need for guided patient selection for CAIX-based therapies.

In addition to direct stimulation of the host immune response, mAbs may be used as a target-specific vehicle for the delivery of therapeutic payloads (Scott et al., 2012). Internalization of mAbs is required for delivery of radioisotopes and cytotoxic drugs to cancer cells, and CAIX mAbs have been developed that exploit this functionality. For example, the cG250 mAb can be internalized by cancer cells (Zatovicova et al., 2014), and treatment of xenograft tumors with radioimmunoconjugates employing the cG250 mAb has demonstrated a delay in growth (Brouwers et al., 2004). Recently, a phase II trial in patients with metastatic clear cell renal carcinoma (mccRCC) treated with ^177^Lu-Girentuximab achieved stable disease in 9 of 14 patients (Muselaers et al., 2015; Table 1), demonstrating the potential utility of such conjugates in CAIX-positive disease. Furthermore, a study in which a novel CAIX mAb was conjugated to the microtubule inhibitor monomethyl auristatin E (MMAE) (BAY79-4620) demonstrated efficacy in several preclinical human xenograft tumor models in which the level of efficacy correlated with the level of CAIX expression (Petrul et al., 2011). A phase 1 clinical trial (NCT01028755) involving the treatment of patients with solid tumors with BAY79-4620 was initiated, but was terminated early due to issues related to patient safety. While the basis for the adverse safety profile of this ADC is not known, recent improvements in ADC technology, including the use of non-cleavable linkers, will certainly provide avenues for continued development of ADCs using CAIX as a target.

In addition to small molecule inhibitors and antibodies, other modalities for targeting CAIX *in vivo* are showing promise. For example, small molecule drug conjugates comprised of an acetazolamide derivative linked to the maytansinoid DM1 were found to accumulate in CAIX-positive lesions and have antitumor effects using the SKRC52 renal cell carcinoma model (Krall et al., 2014). Such conjugates have the advantage of increased access to tumor tissue while enabling delivery of potent cytotoxic compounds (Wichert and Krall, 2015) and engineering of conjugates using current, highly CAIX-specific small molecules may prove effective. Interestingly, a dendritic cell (DC) based vaccine designed to use CAIX as a tumor associated antigen for immune targeting where DCs are engineered to express the GM-CSF-CAIX (AdGMCAIX) fusion protein has been developed. This vaccine has demonstrated preclinical success in a model of RCC when treated in an immunization or intervention setting (Birkhäuser et al., 2013). Based on the recent FDA approval of the DC based vaccine Sipuleucel-T and with other vaccines in phase III evaluation (Palucka and Banchereau, 2013), it will be interesting to see how the AdGMCAIX vaccine progresses clinically as a trial has been initiated (Table 1) and is recruiting patients to test this vaccine in metastatic RCC (NCT01826877). Together, these therapeutic strategies provide a robust platform for targeted treatment of hypoxic tumors in patients.

### MCT1 and MCT4

Cancer cells relying on glycolysis to support survival and rapid proliferation in hypoxia produce large amounts of acidic byproducts, particularly lactate (Gatenby and Gillies, 2008; Gillies et al., 2012; Marchiq and Pouysségur, 2015; Parks et al., 2015). The production of lactate contributes to intracellular acidosis, a situation requiring an adaptive response to increase lactate efflux and potentiate survival (Parks et al., 2015). Cancer cells enhance the efflux of lactate through the upregulation of members of the MCT family of lactate/H^+^ symporters (Doherty and Cleveland, 2013). Two members of this family, MCT1 and MCT4, are upregulated in several cancers, including breast, colorectal, lung, kidney, and glioblastoma (Doherty and Cleveland, 2013; Doyen et al., 2014), and function to regulate lactate transport across the plasma membrane (Halestrap, 2013; Marchiq and Pouysségur, 2015). The differential distribution of MCT1 and MCT4 in cancer cells highlights their distinct roles in lactate transport and demonstrates cooperativity between the two transporters. MCT4 is upregulated in hypoxia as a direct target of HIF-1α and functions to export lactate from hypoxic tumor cells (Doherty and Cleveland, 2013; Parks et al., 2015). Importantly, MCT-mediated extrusion of lactate contributes to acidosis of the TME and plays a role in tumor cell migration and invasion, angiogenesis and immunosuppression (Marchiq and Pouysségur, 2015; Figure 1). MCT1, in contrast, is expressed on oxidative tumor cells and functions to import lactate to feed the TCA cycle through conversion to pyruvate, forming a lactate shuttle and engaging a process termed “metabolic symbiosis” (Payen et al., 2015b). The reliance of hypoxic tumor cells on MCT function to adapt to the potentially detrimental consequences of acidosis and the cooperativity that exists between MCT1 and MCT4 in regulating lactate levels in hypoxia has opened the door to targeting both of these transporters for cancer therapy.

Significant efforts are currently underway to target the MCTs with small molecule inhibitors. While the first generation MCT inhibitors were not clinically viable, owing to a lack of MCT specificity and associated toxicity (Marchiq and Pouysségur, 2015), a potent, second generation MCT1 inhibitor from AstraZeneca, AZD3965, has shown anticancer effects in a variety of cancer cell lines (Bola et al., 2014; Polanski et al., 2014) and treatment of tumors *in vivo* reduced tumor growth and increased sensitivity to radiation (Bola et al., 2014). AZD3965 is undergoing phase 1 clinical trials (NCT01791595) for solid tumors and diffuse large B cell lymphoma (Marchiq and Pouysségur, 2015; Table 1). There have also been efforts to target CD147/Basigin, a transmembrane glycoprotein that functions as a chaperone for folding and trafficking of MCT1 and MCT4 to the plasma membrane (Doherty and Cleveland, 2013; Marchiq and Pouysségur, 2015). Genetic depletion of CD147, when coupled to MCT1 and MCT4, reduced the growth of colon carcinoma tumors (Le Floch et al., 2011). Directed targeting of CD147 has focused on antibody-based therapeutics and mAbs against CD147 have shown efficacy in preclinical cancer models. However, studies have shown that functional redundancy exists between MCT1 and MCT4, and genetic silencing or pharmacological inhibition of MCT1 in human colon adenocarcinoma cells was effective only when combined with MCT4 depletion (Le Floch et al., 2011; Marchiq and Pouysségur, 2015). These results suggest that combinatorial targeting of MCT1 and MCT4 may be required to elicit a robust therapeutic response. It has also been suggested that a limitation of targeting MCTs may be the potential for on-target toxicity in normal tissues and dose limiting side effects in humans due to their ubiquitous expression and involvement in multiple functions, including metabolism, pH regulation, angiogenesis and the immune response (Marchiq and Pouysségur, 2015). However, despite these potential challenges, emerging data demonstrates the potential power of targeting MCT4, particularly for hard-to-treat cancers.

Upregulated MCT4 expression has been reported in human breast cancer, with especially high levels in aggressive, triple negative breast cancer cells that correlated with decreased overall survival (Doyen et al., 2014). MCT4 was also found to be a key regulator of breast cancer cell metabolism and survival in an unbiased, functional RNAi screen and silencing its expression reduced glycolytic flux, increased dependence on oxidative phosphorylation and glutamine metabolism, and reduced spheroid growth (Baenke et al., 2015). It has also been shown recently that MCT4 is over-expressed in a glycolytic subtype of pancreatic cancer and its depletion in xenograft models significantly impacted tumor metabolism and rapid tumor growth (Baek et al., 2014). To date, inhibitors specific for MCT4 have not become available, although one report has suggested that AstraZeneca is currently testing a potent, specific MCT4 inhibitor (Marchiq and Pouysségur, 2015). It remains to be seen whether co-targeting of MCT1 and MCT4 in the context of hypoxia will provide therapeutic benefit. However, together with CAIX, MCT4 plays an important role in the maintenance of glycolytic flux and pH regulation in hypoxic tumor cells and suggests that cotargeting of MCTs and CAIX may serve to further limit the growth of hypoxic solid tumors.

## Targeting angiogenesis

Inhibition of vascularization by treatment with inhibitors of angiogenesis, generally termed anti-angiogenic agents, thereby effectively starving tumor cells of nutrients and oxygen, is a clinically validated strategy for cancer therapy (De Bock et al., 2011; Jain, 2014). However, what was seen initially as a panacea for cancer patients has fallen short of expectations. In particular, many patients fail to respond to anti-angiogenic therapy and those who do often show only a modest survival benefit (Rapisarda and Melillo, 2012; McIntyre and Harris, 2015). While vascular “normalization” can occur and leads to reduced hypoxia and interstitial pressure in the TME, thereby increasing perfusion and improving delivery of chemotherapeutic agents (Jain, 2014; McIntyre and Harris, 2015), the most frequent response to anti-angiogenic therapy is vascular regression and vessel pruning, resulting in increased intratumoral hypoxia (Rapisarda and Melillo, 2012; McIntyre and Harris, 2015), as assessed by hypoxic gene signatures and increased expression of hypoxia-induced effectors, including CAIX.

Many patients are innately resistant to anti-angiogenic agents or rapidly develop acquired resistance in response to treatment. Amongst several resistance mechanisms that have been identified to enable cancer cells to circumvent angiogenesis blockade (van Beijnum et al., 2015), the development of increased intratumoral hypoxia as a result of extensive vessel pruning is of critical importance (Rapisarda et al., 2009; Hu et al., 2012; McIntyre et al., 2012; Rapisarda and Melillo, 2012; Kim et al., 2013; McIntyre and Harris, 2015). Treatment with anti-angiogenic agents has resulted in increased invasiveness and consequently metastases preclinically (Ebos et al., 2009; Pàez-Ribes et al., 2009). However, at least one retrospective study suggests that this is not the case in the clinical setting, although the patients investigated often had metastatic disease at the time of treatment initiation (Miles et al., 2011). In addition, it was recently demonstrated that this difference between the preclinical and clinical realms was likely the result of patients being treated with chemotherapy in combination with anti-angiogenic agents, as this combination tested preclinically eliminated the increased metastasis observed from treatment with anti-angiogenic agents alone (Paez-Ribes et al., 2015).

Since the hypoxia induced by anti-angiogenic therapy stimulates adaptations by the tumor cells that promote therapeutic resistance, it has been suggested that these adaptations may now be critical for survival, producing a type of synthetic lethality termed “induced essentiality” (McIntyre and Harris, 2015). Targeting critical downstream effectors of hypoxia, or HIF-1α itself, has provided a therapeutic advantage in preclinical models (Rapisarda et al., 2009; Hu et al., 2012; McIntyre et al., 2012; Kim et al., 2013). Furthermore, as a consequence of treatment induced hypoxia, CAIX expression is significantly upregulated, suggesting that targeting CAIX in combination with anti-angiogenic agents may provide an effective therapeutic strategy (Hu et al., 2012; Kim et al., 2013). McIntyre and colleagues provided proof of principle data for this co-targeting strategy by showing that genetic depletion of CAIX in combination with bevacizumab in models of colorectal cancer and glioblastoma resulted in a significant delay in tumor growth (McIntyre et al., 2012). Similar results were observed upon treatment with the broad spectrum carbonic anhydrase inhibitor, acetazolamide. Acetazolamide was also utilized recently in combination with bevacizumab in a model of cholangiocarcinoma. While bevacizumab alone showed considerable efficacy in this model, the combination of bevacizumab with acetazolamide further delayed tumor growth (Vaeteewoottacharn et al., 2016). However, the lack of specificity of acetazolamide is a significant caveat of these studies, and investigation of therapeutic combinations using inhibitors specific for CAIX, such as SLC-0111, should be undertaken. In addition to VEGF antibodies, tyrosine kinase inhibitors have been utilized to block angiogenic signaling at the level of the cellular receptors. Treatment of patients with metastatic clear cell renal cell carcinoma with sunitinib results in objective response rates of 40% (Molina et al., 2014). Combinatorial treatment with the CAIX antibody, cG250/Girentuximab, in combination with sunitinib was tested clinically in patients with metastatic RCC (NCT00520533). Unfortunately, the trial ended early due to toxicity issues. Based on preclinical studies, it is clear that this is an area that warrants careful evaluation clinically with rational therapeutic combinations targeting treatment induced hypoxia.

The hypoxic niche of solid tumors is an environment that drives aggressive tumor cell behavior and is a known source of cancer stem cells (CSCs; Currie et al., 2013). As conventional chemotherapy and radiation treatment are known to be less effective in this microenvironment, rational drug combinations need to be developed in order to effectively overcome treatment failure due to therapy induced adaptations. Interestingly, sunitinib and bevacizumab treatment of breast cancer xenografts was demonstrated to increase intratumoral hypoxia and the population of stem cells found within the tumor (Conley et al., 2012). Therefore, it is conceivable that combining drugs that increase intratumoral hypoxia with a drug that can effectively eradicate the CSC pool in the hypoxic niche, such as CAIX inhibitors, may overcome treatment resistance. Alternatively, therapeutic initiatives aimed at promoting re-oxygenation of tumor tissues, including vessel normalization strategies (Jain, 2014) and the use mild hyperthermia (Moon et al., 2010; Datta et al., 2015) in combination with radiotherapy or chemotherapy are proving successful in the clinic. Recent technological advances in the delivery of hyperthermia as a radio- and chemosensitizer, together with the relative absence of additional significant toxicity, have reinvigorated efforts using thermoradiotherapy and thermochemotherapy (Moon et al., 2010; Datta et al., 2015) as combinatorial treatment strategies for hypoxic, solid tumors.

## Targeting immune dysfunction

The cellular composition of solid tumors consists of many stromal cell types in addition to the epithelial cancer cells (Gabrilovich et al., 2012). There exists an extensive communication network between the stroma and the tumor that ultimately results in the hijacking of the stroma by the tumor supporting disease progression, increased invasiveness and enhanced metastatic propensity, angiogenesis, therapeutic resistance and resistance to immune cell eradication mechanisms (Gabrilovich et al., 2012; Quail and Joyce, 2013). The contribution of the various stromal cell types to the tumor have been reviewed extensively elsewhere (Hanahan and Coussens, 2012; Junttila and de Sauvage, 2013). Here, we will focus on three very prominent stromal cell types that mediate immunosuppression in the hypoxic TME and allow the tumor to escape immune detection: myeloid-derived suppressor cells (MDSC), regulatory T cells (Treg) and tumor associated macrophages (TAMs). We will discuss the various mechanisms by which these cell types contribute to tumor progression and therapeutic resistance in the context of hypoxia, as well as the efforts to target the immunosuppressive functions of these cell populations, especially using immune checkpoint inhibitors.

### Myeloid-derived suppressor cells

**Figure 2:**
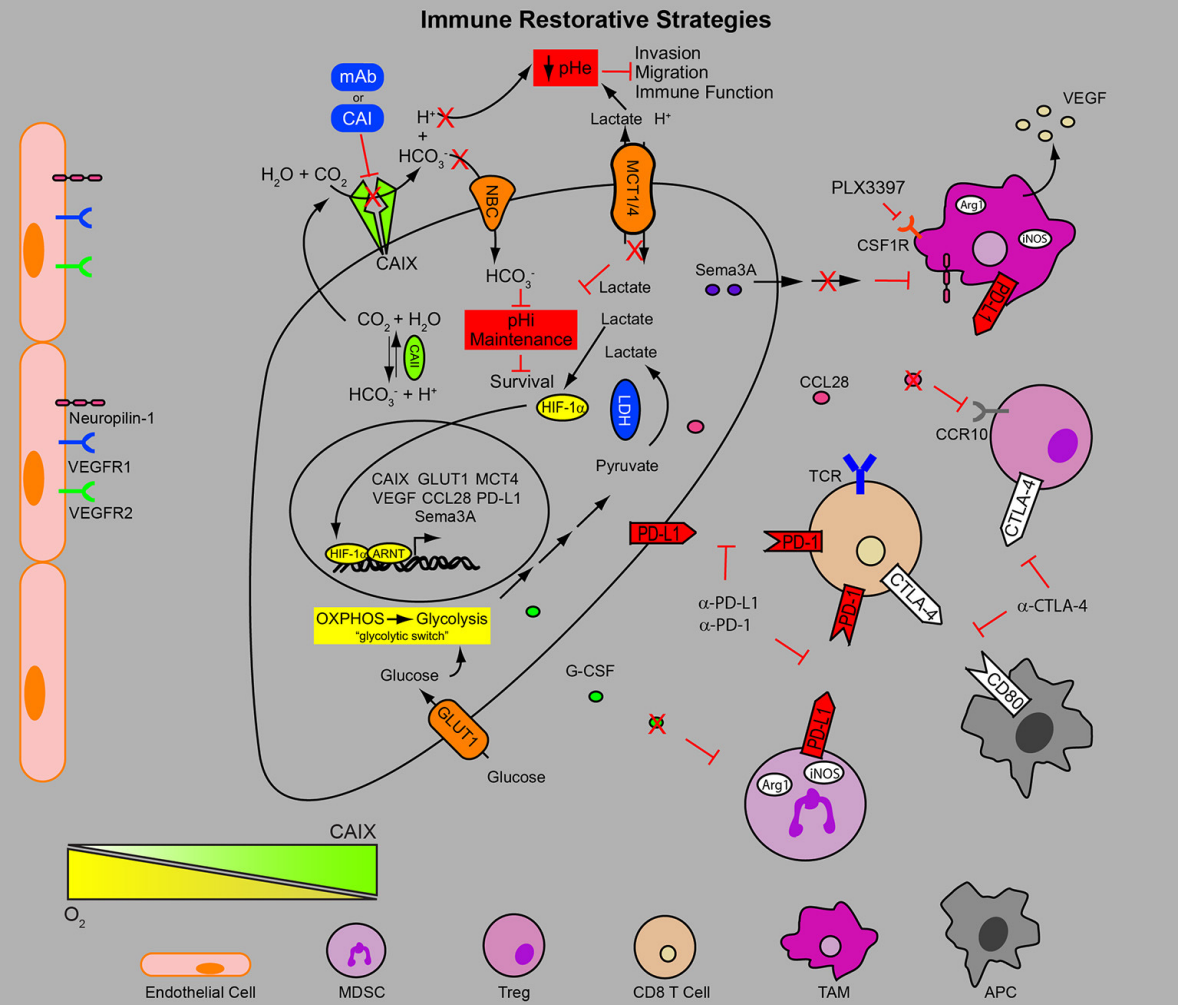
**Combinatorial approaches to target the hypoxic TME and immune dysfunction**. In addition to the metabolic adaptations initiated by engagement of the HIF program in the hypoxic niches of the tumor contributing to the migratory and invasive phenotype of the cancer cells, metabolic byproducts (e.g., low pH) reduce anti-tumor immune function. Furthermore, many HIF target genes (e.g., VEGF, Sema3A, CCL28) secreted by the tumor result in chemotaxis of immune suppressive cell populations (MDSC, TAM, and Treg), limiting the function of anti-tumor immune response. In addition HIF-1-mediated upregulation of PD-L1 by the tumor, MDSC and TAM populations provides an additional layer of resistance to immune surveillance mechanisms. Consequently the use of checkpoint inhibitors to restore anti-tumor immunity to eradicate the therapy resistant cells in the hypoxic TME in combination with inhibitors of pH regulation and recruitment of immune suppressive stromal cell populations may circumvent many of the hurdles facing anti-tumor immunity. pHe, extracellular pH; pHi, intracellular pH; mAb, monoclonal antibody; CAI, carbonic anhydrase inhibitor; ${\text{HCO}}_{3}^{-}$, bicarbonate; H^+^, proton; CO_2_, carbon dioxide; H_2_O, water; NBC, sodium/bicarbonate cotransporter; MCT1/4, monocarboxylate transporter 1 and 4; HIF-1α, hypoxia-inducible factor 1 alpha; ARNT, aryl hydrocarbon receptor nuclear translocator; LDH, lactate dehydrogenase; OXPHOS, oxidative phosphorylation; CAII, carbonic anhydrase II; CAIX, carbonic anhydrase IX; GLUT-1, glucose transporter 1; Sema3A, semaphorin 3A; VEGF, vascular endothelial growth factor; CCL28, chemokine (C-C motif) ligand 28; CCR10, chemokine (C-C motif) receptor 10; Arg1, arginase 1; iNOS, inducible nitric oxide synthase; CSF1R, colony stimulating factor 1 receptor; TCR, T cell receptor; G-CSF, granulocyte colony stimulating factor; TAM, tumor associated macrophage; MDSC, myeloid-derived suppressor cell; Treg, regulatory T cell; APC, antigen presenting cell; VEGFR1, vascular endothelial growth factor receptor 1; VEGFR2, vascular endothelial growth factor receptor 2; CTLA-4, cytotoxic T lymphocyte antigen-4; PD-1, programmed cell death receptor 1; PD-L1, programmed cell death 1 ligand.

Myeloid-derived suppressor cells (MDSC) are an immature myeloid cell population named for their potent immunosuppressive ability toward T and NK cells (Bronte, 2009; Gabrilovich and Nagaraj, 2009) which play a prominent role in tumor progression (Talmadge and Gabrilovich, 2013). In healthy individuals this population is present in very small numbers in the bone marrow, lacks immunosuppressive activity and readily differentiates into mature macrophages and neutrophils. However, in mice and patients with cancer this differentiation is diverted by the abnormally high levels of tumor-derived myeloid growth factors in the blood (Messmer et al., 2015) and circulating MDSC levels have been shown to correlate with increased disease burden in patients with cancer (Diaz-Montero et al., 2009; Messmer et al., 2015). Recruitment of MDSC to tumors has been shown to occur in response to multiple soluble factors, although the precise chemotactic factors involved vary depending on the tumor model interrogated (Acharyya et al., 2012; Gabrilovich et al., 2012; Wesolowski et al., 2013; Highfill et al., 2014; Palazon et al., 2014). Together with these soluble factors, tumor hypoxia, via HIF-1α stabilization and the production of downstream effectors, plays a role in facilitating MDSC mobilization into the circulation and recruitment to tumors (Du et al., 2008; Erler et al., 2009; Wong et al., 2011; Sceneay et al., 2012; Chafe et al., 2015; Figure 2), and regulates the immunosuppressive functions of MDSC (Corzo et al., 2010). Recently, hypoxia was further implicated in expanding the immunosuppressive arsenal of MDSC through the HIF-1 mediated upregulation of programmed cell death 1 ligand 1 (PD-L1; Noman et al., 2014). Moreover, the response to hypoxia has been shown to impact MDSC by triggering rapid differentiation into TAM (Corzo et al., 2010). The same study also identified increased F4/80 (macrophages) positivity relative to Gr1 (MDSC) in hypoxic, pimonidazole staining regions of tumors, suggesting that TAM arising from MDSC may be vitally important for tumor progression in these niches.

MDSC suppress the anti-tumor immune response (Gabrilovich et al., 2012), therapeutic efforts to restore this response (Highfill et al., 2014; Kim et al., 2014), as well as limit the efficacy of anti-angiogenic (Shojaei et al., 2007, 2009) and chemotherapies (Acharyya et al., 2012). Consequently, a number of therapeutic approaches to target MDSC are being explored which can be grouped into 4 distinct categories: (1) forced differentiation (2) targeting soluble factors mediating expansion and/or recruitment (3) direct targeting with the use of chemotherapy (4) reducing their immunosuppressive capacity. Each of these therapeutic strategies has been reviewed extensively in Wesolowski et al. (2013) and targeting MDSCs using immune checkpoint inhibitors is discussed below.

### Regulatory T cells

Regulatory T cells (Treg) are another important stromal cell population that supports tumor progression by contributing to immune evasion (Facciabene et al., 2012). Similar to MDSC, Treg are normally required to keep the immune system tightly regulated and prevent autoimmunity. Treg are found in one of two forms: natural Treg (nTreg) which are thymically derived, or induced Treg (iTreg) which can be induced from naïve CD4 T cells. The fate and function of these populations are controlled by the expression of the FoxP3 transcription factor (Facciabene et al., 2012). Treg have been identified in many cancers where they have been associated with worse prognosis (Zou, 2006; Facciabene et al., 2012).

Like MDSC, Treg have a number of weapons to reduce anti-tumor immunity such as the secretion of immunosuppressive cytokines, direct killing of effector cells, increasing local pools of the toxic metabolite adenosine and disruption of dendritic cell function (Facciabene et al., 2012). Hypoxia-induced CCL28 secretion by the tumor has been shown to induce the CCR-10-dependent recruitment of Treg to tumors (Figure 2), exacerbating the immunosuppressive pressure that allows the tumor to evade host destruction (Schlecker et al., 2012). It has also been shown that Treg are important for the formation of new blood vessels in the tumor (Facciabene et al., 2011). Importantly, these cells can be targeted by immune checkpoint inhibitors such as ipilimumab (anti-CTLA-4; Selby et al., 2013).

### Tumor associated macrophages (TAM)

Tumor-associated macrophages make up the largest population of stromal cells within growing solid tumors and while they are generally described as belonging to the classically activated M1 or the alternatively activated M2 phenotype, these cells actually display a great degree of phenotypic plasticity (Noy and Pollard, 2014). The M1 phenotype is tumoricidal and pro-inflammatory, whereas the M2 phenotype is pro-tumoral and suppresses the inflammatory response (Noy and Pollard, 2014). The recruitment of macrophages to tumors is largely dependent upon soluble factors released into the circulation by the tumor, including CCL2, CCL5, VEGF, Endothelins, endothelial monocyte activating polypeptide (EMAP) II and colony stimulating factor 1 (CSF1), resulting in the mobilization of monocytes from the bone marrow (Murdoch et al., 2004; Franklin et al., 2014). Once macrophages reach the tumor, migration to the hypoxic regions is driven by VEGF, EMAPII, Endothelin-2, CXCR4 and Semaphorin3A (Murdoch et al., 2004; Casazza et al., 2013; Figure 2). While tumor hypoxia was thought to play a prominent role in polarizing TAMs, recent findings suggest that tumor hypoxia merely enhances the expression of pro-angiogenic and metastasis promoting genes (Laoui et al., 2014). TAMs expressing M2 markers were found in the same proportion in well oxygenated and poorly oxygenated tumors in a model of PHD2 haploinsufficiency (Laoui et al., 2014). However, immunosuppressive M2 macrophages are found in greater number in hypoxic regions of solid tumors compared to M1 macrophages (Murdoch et al., 2004; Movahedi et al., 2010; Laoui et al., 2014), supporting a role for hypoxia in M2 TAM chemotaxis and retention in these niches. Hypoxic TAMs provide a rich source of VEGF, augmenting tumor angiogenesis, blood vessel dysfunction and exacerbation of the hypoxia (Stockmann et al., 2008; Figure 2). VEGF secreted by macrophages is also responsible for a role of macrophages in assisting the transit of migratory tumor cells into the vasculature to facilitate their entrance into the circulation (Harney et al., 2015). These macrophages, together with the migratory cancer cells and the endothelial cells, form the “tumor microenvironment of metastasis” (TMEM). The TMEM has been observed in the tumors of breast cancer patients where it has been shown to be predictive of metastatic potential in ER^+^HER2^−^ cases (Rohan et al., 2014).

The highly immunosuppressive nature of macrophages poses a significant hurdle to anti-tumor immunity especially in hypoxic regions of tumors and TAM presence has been shown to impact breast cancer patient survival (Doedens et al., 2010; DeNardo et al., 2011; Casazza et al., 2013). The stabilization of HIF-1α is required for the immunosuppressive activity of macrophages by increasing the expression of Arg1 and iNos activity (Doedens et al., 2010). Therefore, eliminating this immunosuppressive population is critical for restoring anti-tumor immunity. Recently it was shown that blocking the recruitment of macrophages using antibody blockade of CSF1 or CSF1R antagonism with the small molecule inhibitor PLX3397, which is being evaluated clinically, resulted in a CD8 T cell dependent reduction in tumor burden in the MMTV-PyMT model (DeNardo et al., 2011). As hypoxia has also been shown to upregulate PD-L1 expression on TAMs, treatment of tumors with high TAM presence with immune checkpoint inhibitors may also restore anti-tumor immunity (Noman et al., 2014).

### Targeting the immune response with checkpoint inhibitors

Recent clinical breakthroughs in targeting tumor immunology through the use of immune checkpoint inhibitors have revitalized interest in the field (Hodi et al., 2010; Brahmer et al., 2012; Topalian et al., 2012; Wolchok et al., 2013). However, despite the significant measurable responses observed clinically, treatment with inhibitors to PD-1 and CTLA-4 have only produced objective responses in 10–28% of patients treated when utilized as a monotherapy (Hodi et al., 2010; Brahmer et al., 2012; Topalian et al., 2012). Achieving an understanding of the mechanisms limiting the response to these antibodies as well as the identification of biomarkers that can be utilized to stratify patients that will respond to this therapy alone or may require a combinatorial approach will improve patient outcome.

In order to achieve activation, a T cell must recognize a peptide antigen presented by a major histocompatibility complex (MHC) molecule on the surface of an antigen presenting cell (APC) or cancer cell via its T cell receptor (TCR). When this is done in the context of appropriate stimulating signals through the engagement of costimulatory molecules (e.g., CD28) with their cognate receptors (e.g., CD80), activation of a T cell occurs (Pardoll, 2012; Mahoney et al., 2015). Fail safe mechanisms exist to regulate the activity of the T cell and the duration of the response through the activity of inhibitory receptors or checkpoint molecules. As an example, molecules such as PD-L1 expressed on the surface of APCs or cancer cells binds to its cognate receptor PD-1. This engagement reduces T cell activation and cytolytic activity (Pardoll, 2012; Mahoney et al., 2015; Topalian et al., 2015). Similarly, the expression of cytotoxic T lymphocyte antigen (CTLA)-4 binds, with higher affinity than CD28, to CD80 and CD86 on APCs achieving the same reduction in T cell activation (Pardoll, 2012; Mahoney et al., 2015; Topalian et al., 2015). This is the basis for immune checkpoint blockade where antibodies such as ipilimumab (α-CTLA-4), pembrolizumab (α-PD-1) and nivolumab (α-PD-1) interfere with the engagement of the inhibitory ligand with the receptor to maximize anti-tumor immunity (Figure 2). However, there are significant hurdles to initiating and maintaining this immune response in various cancers, which include the presence of a hypoxic, acidic tumor microenvironment (Kareva and Hahnfeldt, 2013; Motz and Coukos, 2013; Palazon et al., 2014; Joyce and Fearon, 2015; Wherry and Kurachi, 2015).

Recently it has been shown that hypoxia contributes to the limited success of an anti-tumor immune response (Barsoum et al., 2014a). Hypoxia triggers the shedding of major histocompatibility complex (MHC) class I chain-related molecule A (MICA), a ligand required for natural killer (NK) cell and effector cell activation, in an ADAM10 dependent manner, contributing to the evasion of immune surveillance mechanisms (Siemens et al., 2008; Barsoum et al., 2011). Furthermore, hypoxia-induced autophagy was demonstrated to limit NK-mediated cell death in models of breast cancer and melanoma due to autophagosome degradation of granzyme B (Baginska et al., 2013). However, it appears that NK cell mediated tumor killing is one of several immune surveillance mechanisms in place in the syngeneic models interrogated as complete tumor regression was not observed upon autophagy inhibition (Baginska et al., 2013). Nevertheless, it is clear that cancer cells in low oxygen become increasingly more difficult for the immune system to eliminate.

It was identified recently that hypoxia, through the action of HIF-1α, increases the expression of PD-L1 by tumor cells and by tumor-infiltrating MDSC (Barsoum et al., 2014b; Noman et al., 2014). In both cases, HIF-1α was found to bind directly to the hypoxia response element (HRE) in the PD-L1 promoter. It has been known for a number of years now that cancer cells within the tumor are capable of expressing PD-L1 in response to interferon-γ, a significant counter measure to the activation of T cells (Dong et al., 2002; Curiel et al., 2003; Pardoll, 2012). Barsoum and colleagues demonstrate *in vitro* that tumor-educated T cells are much less capable of eliciting a cytolytic effect on hypoxic cancer cells in a PD-L1 dependent manner (Barsoum et al., 2014b). However, preclinical studies in multiple tumor models where antibodies to PD-L1, PD-1 and CTLA-4 are used as a monotherapy have not achieved much success (Grosso and Jure-Kunkel, 2013; Highfill et al., 2014; Kim et al., 2014; Guo et al., 2015; Hu-Lieskovan et al., 2015; Ngiow et al., 2015). Recent success has been achieved by depleting MDSC in combination with checkpoint inhibitors (Highfill et al., 2014; Kim et al., 2014). Depletion through the use of epigenetic modifiers 5-azacytidine and entinostat, a combination currently being explored clinically (Kim et al., 2014) (NCT01928576), in models of colorectal and breast cancer achieved significant anti-tumor effects. A similar enhancement was observed upon blocking MDSC recruitment through CXCR2 antagonism in a model of sarcoma (Highfill et al., 2014). Both studies achieved significant enhancement of immune checkpoint inhibitor efficacy as a result of removing multiple layers of immune suppression within the TME. Since hypoxia is known to recruit MDSC to tumors and induce their immunosuppressive behavior it is intriguing to explore hypoxia driven therapies that may limit the recruitment of MDSC to the tumor. Indeed, CAIX was shown to be required for mobilization of MDSC in an implantable model of breast cancer in a G-CSF-dependent manner facilitating establishment of a breast cancer pre-metastatic niche (Chafe et al., 2015). Thus, targeting CAIX in combination with checkpoint inhibitors may prove effective in this regard.

## Hypoxia-independent mechanisms for CAIX induction

Although hypoxia is arguably the major pathophysiological stimulus for HIF-1-mediated upregulation of CAIX in solid tumors, several alternative “provocateurs” present in the TME may contribute to its induction. For example, it has been demonstrated that lactate promotes HIF-1α accumulation in a hypoxia-independent manner in tumor cells through the inhibition of proline hydroxylation, driving expression of downstream effectors such as VEGF (Lu et al., 2002; De Saedeleer et al., 2012), and, potentially, CAIX. The common chemotherapy drugs paclitaxel, gemcitabine and carboplatin have been recently shown to induce HIF-1α expression in various models of breast cancer in a reactive oxygen species dependent manner (Samanta et al., 2014; Lu et al., 2015). Combination treatments of tumor xenografts with paclitaxel or gemcitabine in combination with the HIF-1α inhibitor digoxin proved more effective than either of the drugs tested as a single agent (Samanta et al., 2014). As the chemotherapy induced expression of HIF-1α, and downstream targets, increased the tumorsphere forming capacity of the basal breast cancer lines tested, it is conceivable that chemotherapy induced HIF-1α stabilization and a concomitant increase in the CSC population is a contributing factor to treatment resistance *in vivo*. However, randomized clinical trials are needed to confirm these preclinical findings. In line with these findings, we have demonstrated in both human and murine breast cancer models that the combination of paclitaxel with the small molecule CAIX inhibitor SLC-0111 significantly delayed tumor growth over either single agent (Lock et al., 2013). Based on the data presented by Samanta et al. (2014) and Lu et al. (2015), our observations may be related to increased CAIX expression in the tumor models tested in response to the chemotherapy induced initiation of the HIF program in a hypoxia independent manner, especially in the CSCs. Furthermore, while paclitaxel and doxorubicin treatment of the MMTV-PyMT model of breast cancer was previously shown to result in increased TAM presence in the tumor, hypoxia was not investigated in this setting (DeNardo et al., 2011). Based on recent findings and the role of tumor hypoxia in TAM recruitment and promotion of the alternatively activated, immunosuppressive phenotype, it would be worthwhile to test inhibitors of HIF targets in combination with the above chemotherapy drugs in this model to prevent TAM enrichment and immune evasion following treatment. It has also recently been demonstrated that estrogen signaling induces HIF-1α expression by direct binding of the estrogen receptor (ER)-α to the estrogen response element (ERE) in the HIF-1α promoter (Yang et al., 2015). HIF-1α expression was associated with resistance to tamoxifen in *in vitro* studies and was correlated with reduced overall survival in ER^+^ patients treated with tamoxifen and chemotherapy, correlating HIF-1α with tamoxifen resistance in patients (Yang et al., 2015). Interestingly, the CAIX promoter was also found to have an ERE and was shown to be induced in rat uteri following estrogen treatment (Yang et al., 2015; Karim et al., 2016). Given the correlation with HIF-1α and tamoxifen resistance, it would be interesting to investigate whether CAIX expression is increased in this patient population. If so, CAIX inhibition might offer a second-line treatment for ER^+^ breast cancer patients that have relapsed on hormone ablation therapy.

## Conclusion

Therapeutic resistance is a critical determinant of cancer patient outcome. Identifying biomarkers of response and determinants of treatment failure is paramount to improving treatment modalities. The acidic pH and low oxygen tension within the hypoxic tumor microenvironment of solid tumors poses a significant hurdle to the efficacy of chemo-, radio- and immunotherapies. The body of work described here points to combinatorial treatment approaches designed to eliminate hypoxic niche cells and improve the response to immunotherapy and chemotherapy. For example, combining immune checkpoint inhibitors such as α-PD-1 or α-CTLA-4 with inhibitors of pH regulatory enzymes such as CAIX and MCT-4 may result in a reduced response to hypoxia while simultaneously stimulating the anti-tumor immune response. Furthermore, the application of CAIX inhibitors in combination with anti-angiogenic agents has the potential to reduce the cancer stem cell compartment in the hypoxic niche that is generated as a byproduct of use of therapies such as bevacizumab and sunitinib. Rational combinatorial approaches such as these may lead to improved clinical trial design and ultimately identify effective regimens to overcome treatment resistance.

## Author contributions

PM, SC, and SD conceived and designed the manuscript. PM and SC drafted the manuscript. PM, SC, and SD critically revised and approved the final manuscript.

## Funding

This work was supported by research grants to SD from the Canadian Institutes of Health Research (CIHR #143318) and the Canadian Cancer Society Research Institute (CCSRI #703191).

### Conflict of interest statement

The authors declare that the research was conducted in the absence of any commercial or financial relationships that could be construed as a potential conflict of interest.
